# Comparative Genome Analysis Reveals an Absence of Leucine-Rich Repeat Pattern-Recognition Receptor Proteins in the Kingdom Fungi

**DOI:** 10.1371/journal.pone.0012725

**Published:** 2010-09-14

**Authors:** Darren M. Soanes, Nicholas J. Talbot

**Affiliations:** School of Biosciences, University of Exeter, Exeter, United Kingdom; Michigan State University, United States of America

## Abstract

**Background:**

In plants and animals innate immunity is the first line of defence against attack by microbial pathogens. Specific molecular features of bacteria and fungi are recognised by pattern recognition receptors that have extracellular domains containing leucine rich repeats. Recognition of microbes by these receptors induces defence responses that protect hosts against potential microbial attack.

**Methodology/Principal Findings:**

A survey of genome sequences from 101 species, representing a broad cross-section of the eukaryotic phylogenetic tree, reveals an absence of leucine rich repeat-domain containing receptors in the fungal kingdom. Uniquely, however, fungi possess adenylate cyclases that contain distinct leucine rich repeat-domains, which have been demonstrated to act as an alternative means of perceiving the presence of bacteria by at least one fungal species. Interestingly, the morphologically similar osmotrophic oomycetes, which are taxonomically distant members of the stramenopiles, possess pattern recognition receptors with similar domain structures to those found in plants.

**Conclusions:**

The absence of pattern recognition receptors suggests that fungi may possess novel classes of pattern-recognition receptor, such as the modified adenylate cyclase, or instead rely on secretion of anti-microbial secondary metabolites for protection from microbial attack. The absence of pattern recognition receptors in fungi, coupled with their abundance in oomycetes, suggests this may be a unique characteristic of the fungal kingdom rather than a consequence of the osmotrophic growth form.

## Introduction

Plants and animals regularly encounter large numbers of potentially harmful micro-organisms, yet the vast majority of these bacteria, fungi, or protozoa are unable to cause disease. The first line of defence for both of these groups of eukaryotes is known as innate immunity [1]. Innate immune defence mechanisms rely on detection of potentially harmful microbes by pattern recognition receptors that are predominately located at the surface of cells. These receptors do not recognise particular pathogens, but rely instead on detecting general features of groups of micro-organisms which have been termed microbe associated molecular patterns (MAMPs). These proteins are sometimes known as pathogen-associated molecular patterns (PAMPs), but are not restricted to micro-organisms capable of causing disease [2]. To be effective, MAMPS should ideally be conserved and essential for the viability of a microbe, so that a potential pathogen cannot evade host defences due to a mutation that leads to loss, or alteration, of the molecule [2]. Examples of bacterial MAMPs include specific components of the cell wall or cell membrane, such as peptidoglycan, lipopolysaccharides or lipoteichoic acid, or conserved proteins such as flagellin and the translation elongation factor EF-Tu [3]. Fungal MAMPs have also been identified, including chitin and beta-glucan components of the fungal cell wall [1]. Pattern recognition receptors identified in both plants and animals possess an extracellular leucine-rich repeat (LRR) domain that recognises a particular MAMP. This 20–29 amino acid sequence motif is involved in protein-protein interactions and forms a repetitive structure that can evolve rapidly, creating new variants [4]. Therefore it is highly suited for involvement in immune recognition. Although both animal and plant pattern recognition receptors have extracellular leucine-rich repeat domains and a single membrane spanning helix, they have different intracellular effector domains, which activate defences in response to the detection of a potential pathogen. Animal pattern recognition receptors, for instance, have intracellular Toll/interleukin-1 receptor (TIR) domains and are known as Toll-like receptors, after the first example found in *Drosophila melanogaster*. At least 11 families of toll-like receptors (TLR) have been discovered in mammals and the MAMP recognised by each TLR has been well characterised [5]. In plants, the intracellular effector domain is often a protein kinase as, for example, in FLS2 in *Arabidopsis thaliana* which recognises bacterial flagellin, though there are examples of plant pattern-recognition receptors that have no characterised motifs in their intracellular domain [2]. The LRR-receptor kinases are part of the receptor-like kinase family in plants, which in *Arabidopsis thaliana* number over 400 proteins [6]. As well as innate immunity, LRR-receptor kinases are also involved in the response to specific pathogens, hormone perception and control of development [7]. Although superficially similar, there is no evidence of an evolutionary relationship between plant LRR-receptor kinases and animal toll-like receptors and therefore they are thought to be the result of convergent evolution [2], [8].

Basal immunity is thought to represent the most basic method for an organism to protect itself against potential microbial pathogens, although both animals and plants also possess more specific immune responses to protect themselves against pathogens that have evolved the ability to circumvent this first line of defence [8], [9]. We were interested to find out whether filamentous, osmotrophic organisms required pattern-recognition receptors. These organisms, which include fungi and oomycetes, grow as polarised thread-like cells called hyphae, which secrete extracellular depolymerising enzymes into the environment to breakdown complex polymers such as cellulose, lignin, polysaccharides and proteins, transporting the resulting simple sugars and amino acids into the growing hyphal network. Osmotrophs may therefore perceive and respond to potential pathogens in a different way in light of their highly distinctive growth habit. It is clear that fungi and oomycetes do, however, succumb to infections caused by viruses and microbial pathogens. For example, the chestnut blight pathogen *Cryphonectria parasitica* is infected by a hypovirus, that alters its behaviour and pathogenicity [10] and bacteria can suppress fungal growth. *Lysobacter enzymogenes* can, for instance, act as a biological control agent for fungal diseases of plants [11]. Furthermore, basidiomycete mushrooms succumb to a wide range of bacterial diseases.

Given the widespread nature of pattern recognition receptor proteins, we decided to test whether basal defence from infection by microbial pathogens occurs via leucine-rich repeat containing pattern recognition receptors across all eukaryotic kingdoms, or alternatively, whether osmotrophic organisms such as fungi and oomycetes have independently evolved alternative strategies for contending with microbial attack.

In this study, we utilised a wide cross-section of available eukaryotic genomic sequence data to investigate the conservation of LRR-receptors among eukaryotic organisms and, in particular, to test for the occurrence of such receptors in a large selection of taxonomically diverse osmotrophic species for which completed genome sequences are available. In this way we have been able to investigate the potential innate immunity mechanisms that occur in the kingdom Fungi and in the distantly related oomycetes.

## Results

### LRR-domain receptors are almost entirely absent in Fungi

**Figure 1:**
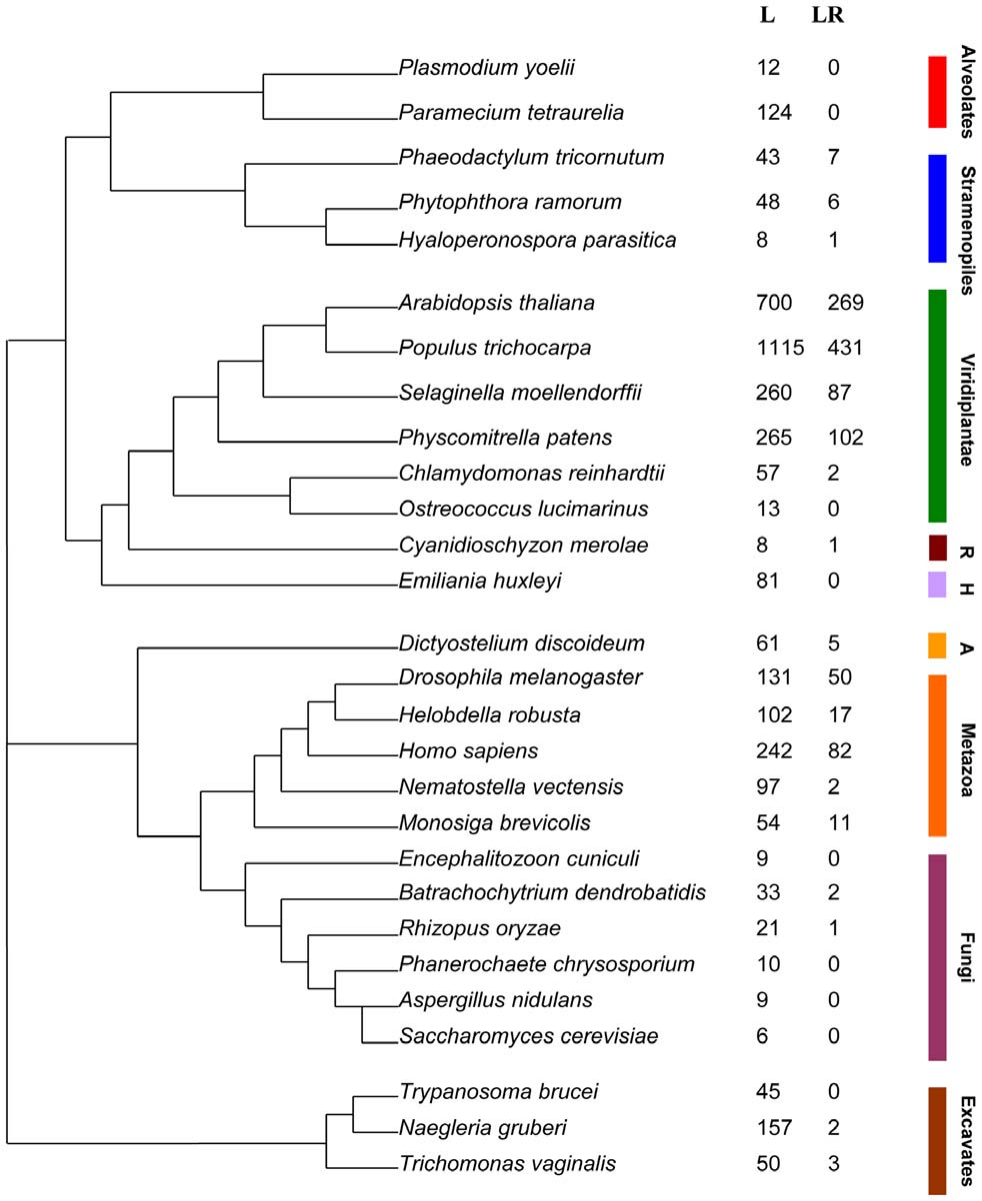
Phylogenetic distribution of LRR domain containing proteins. Species tree showing the numbers of LRR-domain (L) and LRR-receptor (LR) encoding genes in the genomes of representative species from each of the taxonomic groups studied. Broad phylogenetic groups are indicated by coloured bars (A =  Amoebozoa, R = Rhodphyta, H = Haptophyte). The tree is schematic and is based on Burki *et al.*, [35].

We set out to investigate the occurrence of leucine-rich repeat (LRR) domain containing receptors throughout the available eukaryotic genomic data sets. The 101 species selected for this study (Table S1), represent all branches of the eukaryotic phylogeny for which genome sequences are publicly available. These include metazoa, plants (both land plants and unicellular chlorophytes), fungi, excavates, alveolates, stramenopiles, amoebozoa, rhodophytes and haptophytes. Because our study concentrated particularly on fungi, 49 of the species chosen were from this Kingdom, representing all branches of the fungal lineage for which genomes are publicly available (microsporidia, chytrids, zygomycota, basidiomycota, ascomycota). Sets of predicted proteins generated from the genome sequences of 101 eukaryotic species were scanned for the presence of the three LRR domains present in the Pfam database (accession numbers PF00560, PF07723, PF07725). These LRR-containing proteins were then scanned for other protein motifs in the Pfam database as well as for the occurrence of transmembrane helices. Default thresholds for significance were used in the Pfam search. These are hand-curated for every family and designed to minimise false positives [12]. We defined an LRR-receptor as a protein that contains at least one LRR domain and a single transmembrane helix. The number of LRR-domain containing proteins and LRR-receptors present in each species is shown in Table S2 and Table S3, respectively. Figure 1 illustrates these results as a phylogenetic tree using selected species from each taxon. Although LRR-domain containing proteins were found in all eukaryotic species studied, the numbers varied widely. LRR-domain proteins are most common in the land plants, but also highly abundant in metazoa. Even within taxonomic groups the number can be quite variable. For example, in the alveolates the number of LRR-proteins varies from 124 in *Paramecium tetraurelia* to just 5 in *Cryptosporidium parvum*. The numbers of LRR-containing proteins encoded by the genomes of fungi are comparatively low, ranging from six in the budding yeast *Saccharomyces cerevisiae* and *Mycosphaerella fijiensis* to 37 in *Postia placenta* (Table S2). In fact, the genome of the brown rot fungus *Postia placenta* seems to have an unusually high number of genes encoding LRR-containing proteins within the fungal lineage. In our study, only five fungal species had genomes containing 20 or more genes encoding LRR-containing proteins and more than half (25 species) had less than ten of these genes within their genomes. Differences between taxonomic groups are even more striking when the number of LRR-receptors encoded by the genome of each species was compared (Table S3). LRR-receptor encoding genes are completely absent from the genomes of the alveolates, the haptophyte *Emiliana huxleyi* and 43 out of the 49 species of fungi in this study. This suggests that within these groups recognition of potential pathogens using LRR-receptors, as has been shown previously in animals and plants, is absent. Within the fungi there are only a few examples of LRR-receptor encoding genes (Table S4) and these are only found in six species. Since LRR-receptor encoding genes are absent in the genomes of the majority of fungal species, we aligned the protein sequences encoded by these genes with other closely related sequences (where available) to look for potential anomalies. Myfi88095 from *Mycosphaerella fijiensis* encodes an LRR-domain containing adenylate cyclase that is predicted to have one transmembrane helix. All fungal genomes studied (apart from the chytrid *Batrachochytrium dendrobatidis*) have one gene encoding an LRR-domain containing adenylate cyclase, but none of them are predicted to have a transmembrane helix. Alignment of fungal adenylate cyclase protein sequences showed that the sequence from *M. fijiensis* had an additional 560 amino acids at the N-terminus compared to closely related sequences, which is likely to be due to an error in the gene model. This region contains the predicted transmembrane helix. CNAG_04363 from *Cryptococcus neoformans* encodes an LRR-domain containing protein which is predicted to have one transmembrane helix, although there are no other closely related fungal sequences. The protein encoded by this gene is predicted to have an LRR N-terminal domain in the middle of the protein. If we assume the position of this domain shows the true N-terminus of the protein, then this gene model also has an incorrect extension at the 5′-end which contains the predicted transmembrane domain. Removing these two genes from the list of fungal LRR-receptors leaves us with just five fungal examples of these genes from four species (the chytrid *Batrachochytrium dendrobatidis*, two zygomycete species and the basidiomycete *Postia placenta*). None of these sequences are predicted to have any other functional domains. There are examples of secreted LRR domain-containing proteins involved in innate immunity, for example, plant polygalacturonase inhibiting proteins [13]. By looking for fungal LRR domain-containing proteins that were predicted to have a signal peptide but no transmembrane regions, we found fifteen potential examples of these from 10 species (Table S5). If these proteins do serve potential roles in innate immunity, then they are certainly not widespread among different fungal taxa. There was no evidence that the predicted have transmembrane regions were due to incorrect gene models.

By far the largest numbers of LRR-receptors are encoded in the genomes of land plants. This is not surprising because it has already been shown that LRR-receptors play a number of signalling roles in plants, including innate immunity, response to specific pathogens, and development. There are also large numbers of LRR-receptors encoded by metazoan genomes [7]. Nearly all species of chlorophyte, stramenopile, rhodophyte, excavate and amoebozoa, for instance, encode LRR-receptors. Therefore all these groups may in theory possess a system of basic immune recognition based on LRR-receptors. As well as extracellular LRR domains, receptors involved in the innate immune response in animals and plants have intracellular effector domains, namely Toll/interleukin-1 receptor (TIR) (Pfam accession PF01582) and protein kinase (PF07714, PF00069) respectively [5], [6]. Toll-like receptors (LRR domain(s), 1 transmembrane helix, TIR domain) and LRR-protein kinase receptors (LRR domain(s), 1 transmembrane helix, protein kinase domain) were identified for each species. The numbers are shown in Table S6 and Table S7, respectively. Toll-like receptors were confined to metazoa (apart from a few examples in land-plants) and LRR-protein kinase receptors were found in all species of land plants but also, interestingly, the choanoflagellate *Monosiga brevicollis* and all species of oomycetes. There are also examples of LRR-protein kinase receptors in some metazoan species. The number of LRR-protein kinase receptors in plants range from 70 in *Selaginella moellendorffii* to 301 in *Populus trichocarpa*, consistent with their importance as receptors in a number of physiological roles including defence against pathogens and development [7]. LRR-kinase receptors are absent in chlorophytes, apart from two examples in *Chlorella NC64A*. Interestingly, we identified Toll-like receptors in most lineages of metazoa, including Ecdysozoa, Lophotrochozoa and Deuterostomia, but not in the choanoflagellate *Monosiga brevicollis*, the placozoan *Trichoplax adhaerens* or the cnidarian *Nematostella vectensis*. To confirm the lack of LRR-kinase receptors in fungi, we identified all fungal genes encoding proteins containing both LRR and protein kinase domains (Table S8). There was no evidence that due to incorrect gene models any of these proteins could in fact have transmembrane regions. This list contains four genes from the chytrid *Batrachochytrium dendrobatidis* and also a group of five closely related genes from species of filamentous ascomcyetes. The sequences of the proteins encoded by these genes show a large degree of homology to bacterial LRR-protein kinases of unknown function. Phylogenetic analysis (Figure S1) and the fact that these genes all lack introns provide preliminary evidence that these genes may have resulted from a horizontal gene transfer from bacteria to fungi.

### LRR-domain containing adenylate cyclases are unique to Fungi

**Figure 2:**
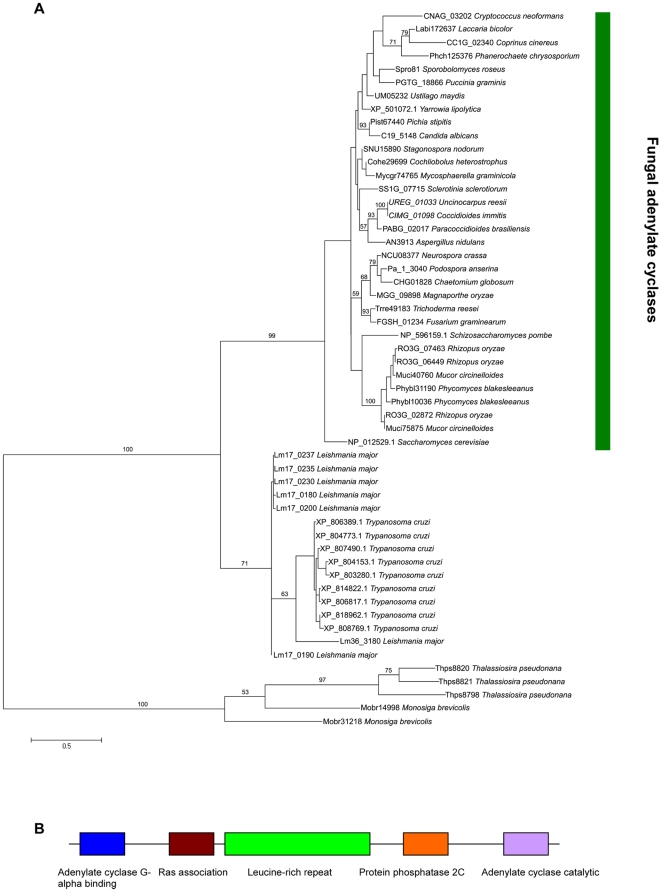
Domain structure and phylogeny of fungal adenylate cyclases. **A** Phylogenetic tree (100 bootstraps) constructed using the adenylate cyclase domain from fungal LRR-domain containing adenylate cyclases and other closely related sequences. These sequences had been identified in a full phylogenetic tree made from adenylate cyclase domains from species in each of the taxonomic groups used in this study (Table S6). Bootstrap scores of 50 or above shown for each branch. Protein IDs (from database sources listed in Table S1) are shown for each taxon. **B** Domain structure of a typical fungal adenylate cyclase. Order of domains shown from N-terminus on left to C-terminus on right. The size of the boxes is not intended to indicate the size of the domains.

As mentioned previously, the number of LRR-domain encoding genes within fungal genomes is less than other eukaryotes sampled. Furthermore, these genes can be related, based on sequence similarity, to genes of known function. These include adenylate cyclase, protein phosphatase 1 regulatory subunit, septation initiation network scaffold protein and glucose-repressible alcohol dehydrogenase transcriptional effector. The structure of adenylate cyclases in fungal species is particularly interesting. Fungi are unique amongst the species studied, in that their genomes contain adenylate cyclase-encoding genes that contain LRR-domains [14]. In fact a survey of genes containing the adenylate/guanylate cyclase catalytic domain (Pfam accession PF00211) in the genomes of the 101 eukaryotic species used in this study revealed no other examples of LRR and adenylate/guanylate cyclase catalytic domains in the same gene. LRR-containing adenylate cyclases were found in the genomes of all species of fungi studied apart from the chytrid *Batrachochytrium dendrobatidis* and the microsporidian *Encephalitozoon cuniculi*. In ascomycota, basidiomycota and zygomycota, every adenylate/guanylate cyclase domain-containing protein also possesses LRR domains. In order to investigate the phylogenetic relationship between adenylate/guanylate cyclase catalytic domain containing proteins, a tree was constructed using sequences from these domains found in proteins from selected species representing all lineages used in this study. The full phylogenetic tree is shown in Figure S2. The sequences representing the adenylate/guanylate cyclase catalytic domain from fungal LRR-containing adenylate cyclases group together with strong branch support (86 bootstraps). The genome of the chytrid *Batrachochytrium dendrobatidis* encodes six proteins containing the adenylate/guanylate cyclase catalytic domain, none of which contain LRR domains. The adenylate/guanylate cyclase catalytic domains from this species do not group with the other fungal taxa, but with sequences from metazoa and amoebozoa, suggesting a totally different evolutionary history. A second tree just using adenylate/guanylate cyclase catalytic domain sequences from LRR-containing adenylate cyclases and the two most closely related outgroups (based on the tree in Figure S2) is shown in Figure 2A. This shows that the most closely related sequences to the fungal adenylate/guanylate cyclase catalytic domains are from the excavates *Leishmania major* and *Trypanosoma cruzi*. These two sets of sequences cluster together with strong branch support (100 bootstraps). It is difficult to provide phylogenetic evidence showing the evolutionary relationship between LRR-domains, due to problems in sequence alignment because of the repetitive nature and high diversity of the sequences [15]. To attempt to do this, we took just the LRR-domain sequences from all the proteins identified in this analysis and clustered them using OrthoMCL. The LRR-domains from fungal adenylate cyclases were all found in the same cluster (ORTHOMCL1). A phylogenetic tree was created using 197 LRR-domain sequences from this cluster (Figure S3). The sequences in ORTHOMCL1 come from most taxa used in this study including both plants and animals. Although the bootstrap supports are not high in many cases, the LRR domains from adenylate cyclases are highly related to each other and most closely related to sequences from *Phytophthora ramorum* and chlorophyte species. None of these closely related sequences contain any other functional domains or transmembrane regions.

The typical domain structure of a fungal adenylate cyclase is shown in Figure 2B. Table S9 shows the domain structure for all fungal LRR-containing adenylate cyclases. The order of the domains from adenylate cyclase G-alpha binding domain (PF08509) at the N-terminus to the adenylate cyclase catalytic domain at the C-terminus is conserved amongst the fungal LRR-containing adenylate cyclases. Some fungal adenylate cyclases lack the G-alpha binding domain (including all those in the species of zygomycota) and a subset of these also do not have the Ras association domain. All of them, however, have the LRR, protein phosphatase 2C and adenylate cyclase catalytic domains. The multi-domain structure of fungal adenylate cyclases likely reflects the complexity of the signalling pathways in which they are involved. In *Saccharomyces cerevisiae*, the adenylate cyclase directly interacts with the alpha subunit of a heterotrimeric G protein and RAS proteins through the G-alpha binding and Ras association domains, respectively [14], [16]. In the rice blast fungus *Magnaporthe oryzae*, the protein phosphatase domain of the adenylate cyclase MAC1 has been shown to interact with both a MAP kinase and serine/threonine kinase [17]. Interestingly, a study has shown that in *Candida albicans*, the adenylate cyclase Cyr1p is involved in detecting the presence of bacteria using the LRR motif [18]. Although Cyr1p is cytoplasmic, muramyl dipeptides (the breakdown products of bacterial cell wall peptidoglycans) appear to enter the cell and directly interact with the LRR domain of this adenylate cyclase inducing a transition from yeast-like to hyphal growth via the cyclic AMP/protein kinase A pathway. Since LRR-containing adenylate cyclases are found in all fungi (apart from chytrids), it would be tempting to hypothesise that they could be involved in bacterial detection in these species as well, but evidence for this is lacking. Many plant resistance gene products act cytoplasmically, detecting pathogenic effectors entering the plants cytoplasm [19]. The largest group of these resistance proteins contain nucleotide binding (NB-ARC, PF00931) and LRR domains (NBS-LRR). We could not identify any fungal proteins that contain both of these domains. Leguminous plants use LysM domain receptor protein kinases to recognise rhizobial bacteria [20], but we found no evidence of genes encoding this type of receptor existing in fungal genomes. We also tried to look for the presence of other domains that are known to be components of pattern recognition receptors in animals [21] in fungal encoded proteins. We found no proteins containing immunoglobin (PF00047), Lectin-C (PF00059), TIR (PF01582), NOD (PF06816), NODP (PF07684), ITAM (PF02189, PF10538), Pyrin (PF02758) or caspase recruitment (PF00619) domains or LRR domains in combination with NACHT (PF05729) domains.

In fungi, cell fusion between genetically unlike individuals triggers cell death, which is known as an incompatibility reaction. It has been proposed that five genes that control this process in the fungus *Podospora anserina* could also be involved in innate immunity [22]. These proteins are part of the STAND family, having an N-terminal cell death effector (HET) domain, a central NACHT domain and a C-terminal WD-repeat domain. They show similarity in domain structure to intracellular animal pattern recognition receptors, except that the animal proteins have a CARD domain at the N-terminus. In order to look at the occurrence of STAND family in the kingdom Fungi, the complete set of predicted proteins from 49 fungal genomes was scanned for NACHT domains and the complete domain structure for each of the NACHT domain proteins predicted (Table S10). NACHT domain proteins are restricted to filamentous ascomycetes. STAND family proteins were found in only four species (*Podospora anserina*, *Cochliobolus heterostrophus*, *Botrytis cinerea*, *Sclerotinia sclerotiorum*), although proteins containing NACHT and WD-repeat domains but not HET domains were found in another 13 species. So if STAND domain proteins were involved in innate immunity, this type of immunity is also restricted to only certain species of filamentous ascomycete.

### Oomycetes possess LRR-receptor kinases

**Figure 3:**
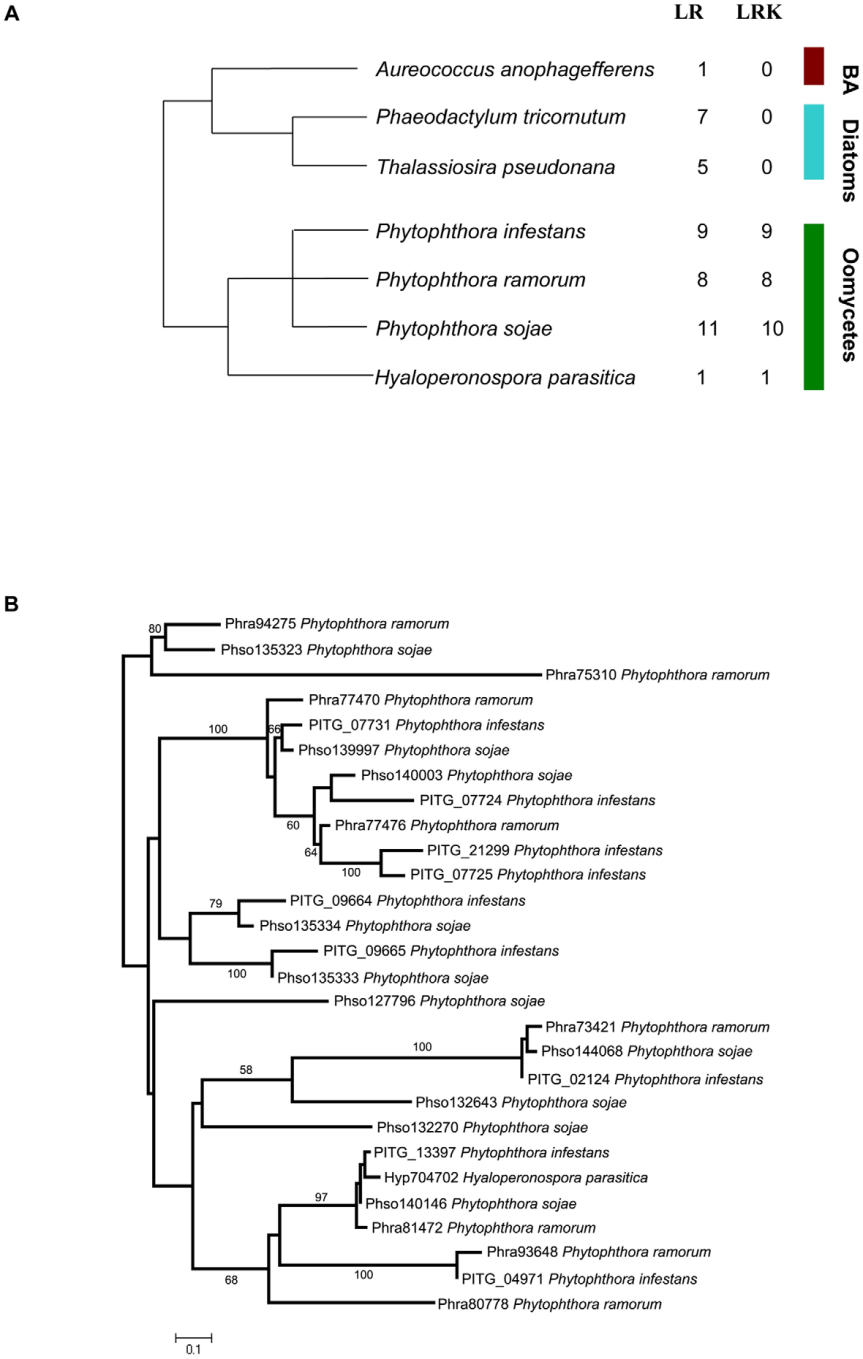
A comparison of LRR-receptors in stramenopiles. **A** Species tree of stramenopiles showing the number of LRR-receptor (LR) and LRR-receptor kinase (LRK) encoding genes in each genome. Broad phylogenetic groups are indicated by coloured bars (BA  =  Brown algae). The tree is schematic and based on Burki *et al.*, [35]. **B** Phylogenetic tree showing LRR-receptor kinases in oomycetes. Bootstrap support scores of 50 or above shown for each branch. Protein IDs (from database sources listed in Table S1) are shown for each taxon.

Analysis of sets of predicted proteins showed that all species of stramenopiles that were analysed encode LRR-receptors in their genomes (Figure 3B). There is, however, a striking difference between the filamentous, osmotrophic oomycetes and the other stramenopiles (the diatoms and brown algae). In oomycetes, every LRR-receptor (with only one exception in *Phytophthora sojae*) also has a protein kinase domain, whereas in the other stramenopile species none of the LRR-receptors have attached functional domains. There also seems to be little sequence similarity, based on BLASTP [23], between LRR-domains from oomycete LRR-receptors and those from other species of stramenopile. By aligning protein sequences encoded by all oomycete genes that contained both LRR and protein kinase domains, we identified an additional two LRR-receptor kinases from *Phytophthora ramorum* that had incorrect gene models where an in-frame intron had been introduced that removed the region containing the transmembrane helix. Figure 3B shows a phylogenetic tree of the oomycete LRR-receptor kinases, showing an example of these hybrid proteins in *Hyaloperonospora parasitica* but also a number of paralogous duplications within the *Phytophthora* lineage. The transmembrane domains in the oomycete LRR-receptor kinases are between the LRR and protein kinase domains, suggesting the latter are likely to be extracellular, as is the case with the plant LRR-receptor kinases [2]. The LRR-domains in the oomycete receptors, however, are much shorter (two LRR repeats) than their counterparts in plants (up to 25 LRR-repeats). Also it seems that in most cases the LRR-domain sequences from oomycete LRR-receptor kinases are more similar, based on BLASTP [23], to sequences encoded by animal genes than those from plants. These results suggest that the oomycetes may possess innate immunity mechanisms that are dependent on LRR-receptor kinases, although it is also possible that LRR receptor kinases serve completely distinct functions in oomycetes, such as control of development.

## Discussion

The innate immunity system in plants and animals involves recognition of microbial specific molecules (MAMPS) by pattern recognition receptors containing extracellular LRR-domains [5], [9]. Given the widespread operation of LRR receptor-based innate immunity mechanisms across both plants and animals, it is easy to assume that innate immunity mechanisms may be ubiquitous among eukaryotes, albeit having evolved by distinct routes [2], [8]. However, prior to this study there has not been a systematic comparative genomics study to address the extent of conservation across eukaryotic organisms. In this project, we analysed the presence of genes that putatively encode LRR-containing proteins across a very wide range of eukaryotic taxonomic groups. We were particularly concerned with whether filamentous, osmotrophic organisms, such as fungi and oomycetes, possessed LRR-receptors, given that their mode of nutrition is so distinct from that of the other eukaryotes. Fungi grow by apical extension and form interwoven networks of thread-like cells called hyphae. They are able to colonise complex and diverse substrates, breaking down polymers by means of extracellular depolymerising enzymes, which they secrete in large amounts from the tips of their polarised hyphae. In this way, fungi break down polysaccharides, proteins, lignin and cellulose and transport in the resulting monomers to sustain their growth and development. These characteristics make fungi and oomycetes very effective pathogens, but it is clear that they must, conversely, encounter potential pathogenic organisms and require a means of perceiving and defending themselves from bacteria and other microbial pathogens, including other fungi. How this mechanism occurs in osmotrophs is also largely unexplored and we therefore set out to explore whether the components of known innate immunity processes were conserved in fungi and oomycetes, or whether they had evolved distinct forms of such pattern recognition receptors.

Our comparative genomics study has shown that classical LRR receptors are largely absent from the kingdom Fungi, being completely absent from 45 of the 49 genome sequences that we sampled. The restricted distribution of LRR-receptor encoding genes in four genome sequences was, furthermore, largely confined to chytrids and zygomycota, with normally only a single example per genome. This is strikingly different from the situation in both plants and animals where dramatic gene family expansion has occurred and this suggests that widespread use of LRR receptors is not a feature of fungi. How then do fungi perceive and respond to microbial pathogens? One clue to how this might occur comes from the recent functional study of the role of LRR domains in the adenylate cyclase protein of *Candida albicans* [18]. It has been demonstrated experimentally that LRR domains within *C. albicans* adenylate cyclase can detect the breakdown products of bacterial peptidoglycan [19]. In this way the authors speculate that *C. albicans* may have the capacity to detect and respond to bacteria, which may be potential pathogens or competitors. In our study we have shown that possession of these unusual LRR-containing adenylate cyclases is uniquely found in fungi and is widespread among diverse fungal species, suggesting that it may constitute a conserved attribute of these signalling proteins within fungi. Adenylate cyclase is responsible for catalysing the synthesis of cyclic AMP from ATP. In fungi, cAMP-dependent protein kinase A signalling pathways play roles in control of morphogenesis and the response to starvation [19]. The potential dual role of adenylate cyclase in perceiving bacterial cell wall components has thus far only been reported in *C. albicans*, but the possession of such domains in adenylate cyclase genes from such a wide group of fungal species suggests that the biological significance of the LRR domains should be explored further.

The large-scale absence of LRR receptors in fungi is, however, also consistent with fungi possessing alternative mechanisms to detect potential microbial pathogens and defend themselves from invasion. Filamentous fungi are well known to produce a wide range of secondary metabolites, some of which have anti-microbial or antibiotic properties, such as the well-known β-lactam antibiotics like penicillin [20]. It has been suggested that secretion of these antibiotics may give fungi a selective advantage in their natural environment in competition with bacteria and other micro-organisms, although the are few direct experimental tests of this well-established idea [20]. Many secondary metabolic pathways in fungi contain enzymes from highly conserved families, such as cytochrome p450s and polyketide synthases. Identification of members of these families in the gene inventory of a particular fungal species is therefore strongly associated with the secondary metabolic potential of a species and the variety of novel metabolites that can be synthesised [24], [25]. This may provide an alternative strategy for preventing microbial attack, but one that acts at a distance from the advancing fungus, or around sites of sporulation or colonisation of substrates. It has also been suggested that members of the STAND gene family, which are involved in heterokaryon incompatibility, may play a role in fungal innate immunity [22], though our studies suggest that STAND genes are restricted to a small number of species of filamentous ascomycetes.

Interestingly, our analysis also showed that all oomycete species in this study possess LRR-receptors with intracellular protein kinase domains that appear similar to those found in plants. This striking contrast with the absence of pattern recognition receptors in fungi, suggests that the osmotrophic growth habit, shared by these distinct organisms [26], is not the principle reason for the distinct inventory of receptors in fungi. However, it is worth noting that oomycete species completely lack polyketide synthase-encoding genes and have considerably fewer cytochrome p450s and non-ribosomal peptide synthases than filamentous fungi [27], and are therefore likely to secrete a much smaller range of secondary metabolites [27], [28]. It is apparent therefore that fungi and oomycetes have adopted distinct strategies for perceiving and responding to potential microbial pathogens. Fungi appear to possess novel receptors, but also have the capacity to utilise secreted antimicrobial secondary metabolites to defend themselves against potential pathogens, whereas oomycetes, with a restricted capability to synthesise secondary metabolites, are reliant on a battery of potential pattern recognition receptors that may well operate in a similar way to those of plant species to facilitate an innate immune response for protection against other microbes. Investigating the function of LRR-receptors in oomycetes and the novel counterparts in fungi experimentally may therefore offer new insight into the wider evolution of innate immunity in the eukaryotes.

## Materials and Methods

### Genome sequences

For each species used in this study, sets of predicted proteins were downloaded from the sources listed in Table S1. The sequences were stored in a bespoke relational database. The IDs identifying each protein were from the source database, except for those proteins download from the Joint Genome Institute, where four or five letter codes representing the species were added to the numerical protein IDs from this database.

### Identification of Pfam motifs

The Pfam-A library from release 23.0 of the Pfam database was downloaded from the Pfam website (ftp://ftp.sanger.ac.uk/pub/databases/Pfam). This library contains 10,340 protein models constructed from manually curated multiple alignments and covers 73% of proteins in UniProt [12]. It was used to analyse the sequences of predicted proteins for all 101 genomes to identify the Pfam motifs that each protein contains. The analysis was performed using the “pfam_scan” perl script (Version 0.5) downloaded from the Pfam website and HMMER software (downloaded from http://hmmer.janelia.org/). Default thresholds were used, which were hand-curated for every family and designed to minimise false positives [12].

### Identification of transmembrane domains

The sequences of predicted proteins for all 101 genomes were analysed for the number and position of transmembrane helices using a local version of TMHMM (downloaded from http://www.cbs.dtu.dk/services/TMHMM/). TMHMM uses a membrane topology prediction algorithm based on a hidden Markov model [29]. TMHMM can falsely predict N-terminal signal peptides as transmembrane helices. To account for this, SignalP [30] was used to predict the occurrence and size of any signal peptides and TMHMM predicted transmembrane helices that overlapped with the predicted signal peptide were removed from the dataset.

### Phylogenetic analysis

Protein sequences were aligned using MUSCLE version 3.6 [31]. Gaps, poorly aligned and highly divergent regions of the resulting multiple sequence alignment were removed using Gblocks [32]. Default parameters were used for both these programs. Maximum likelihood phylogenies were estimated from the curated alignment with PhyML [33] using the following parameters: protein substitution model  =  WAG, number of substitution rate categories  = 4, gamma distribution parameter  =  estimated, proportion of invariable sites  =  estimated, 100 bootstraps.

### Clustering of leucine-rich repeat domain sequences

The protein sequence containing the leucine-rich repeat domain was extracted for each protein. These 8,001 sequences were clustered into groups of similar sequences using OrthoMCL [34] (e-value cut-off 10^−15^, inflation value 1.5), producing 329 clusters and 3,562 singletons.

### Checking for contamination of genome sequence information

To rule out the possibility that any sequences of interest came from contaminating bacterial or plant DNA, rather than the species for which a given genome had been sequenced, the DNA sequence of all fungal and oomycete LRR-containing genes was compared to the genome sequences of 1,337 species of bacteria and three species of higher plant using BLASTN [23]. No evidence of contamination was found.

## Supporting Information

Figure S1 Phylogenetic tree constructed using sequences that show the greatest homology to a set of LRR-protein kinases found specifically in a few species of filamentous ascomycetes. Bootstrap support scores of 50 or above shown for each branch. Taxa are labelled with protein IDs (for sequences analysed in this project) or GI numbers for other sequences from NCBI database. Fungal sequences are indicated by a blue bar. Note: CHG08425 from Chaetomium globosom has an LRR domain, but lacks a protein kinase domain.
(2.64 MB TIF)

Figure S2 Phylogenetic tree constructed using adenylate/guanylate cyclase domains from representative species in each of the taxonomic groups used in this study. Bootstrap support scores of 50 or above shown for each branch. Protein IDs (from database sources listed in Table S1) are shown for each taxon. Fungal LRR-domain adenylate cyclases are indicated by a blue bar.
(4.46 MB TIF)

Figure S3 Phylogenetic tree constructed using LRR-domain sequences from ORTHOMCL1 cluster (see text for details). Bootstrap support scores of 50 or above shown for each branch. Protein IDs (from database sources listed in Table S1) are shown for each taxon. LRR-domains from fungal adenylate cyclases are indicated by a blue bar.
(0.86 MB TIF)

Table S1 Species used in this study
(0.02 MB XLS)

Table S2 The number of LRR-domain containing proteins identified in each species
(0.02 MB XLS)

Table S3 The number of LRR-receptors identified in each species
(0.02 MB XLS)

Table S4 LRR domain containing receptors in fungi
(0.02 MB XLS)

Table S5 LRR domain proteins containing a signal peptide in fungi
(0.02 MB XLS)

Table S6 The number of Toll-like receptors identified in each species
(0.02 MB XLS)

Table S7 The number of LRR-receptor kinases identified in each species
(0.02 MB XLS)

Table S8 LRR kinase proteins in fungi
(0.02 MB XLS)

Table S9 Protein domain structure of fungal LRR-domain containing adenylate cyclases
(0.04 MB XLS)

Table S10 Protein domain structure of fungal NACHT domain containing proteins
(0.05 MB XLS)
