# Supplementary figures and images for: Comparative Genome Analysis Reveals an Absence of Leucine-Rich Repeat Pattern-Recognition Receptor Proteins in the Kingdom Fungi
Source: PLoS One. 2010 Sep 14;5(9):e12725. doi: 10.1371/journal.pone.0012725 (PMC2939053; doi:10.1371/journal.pone.0012725)

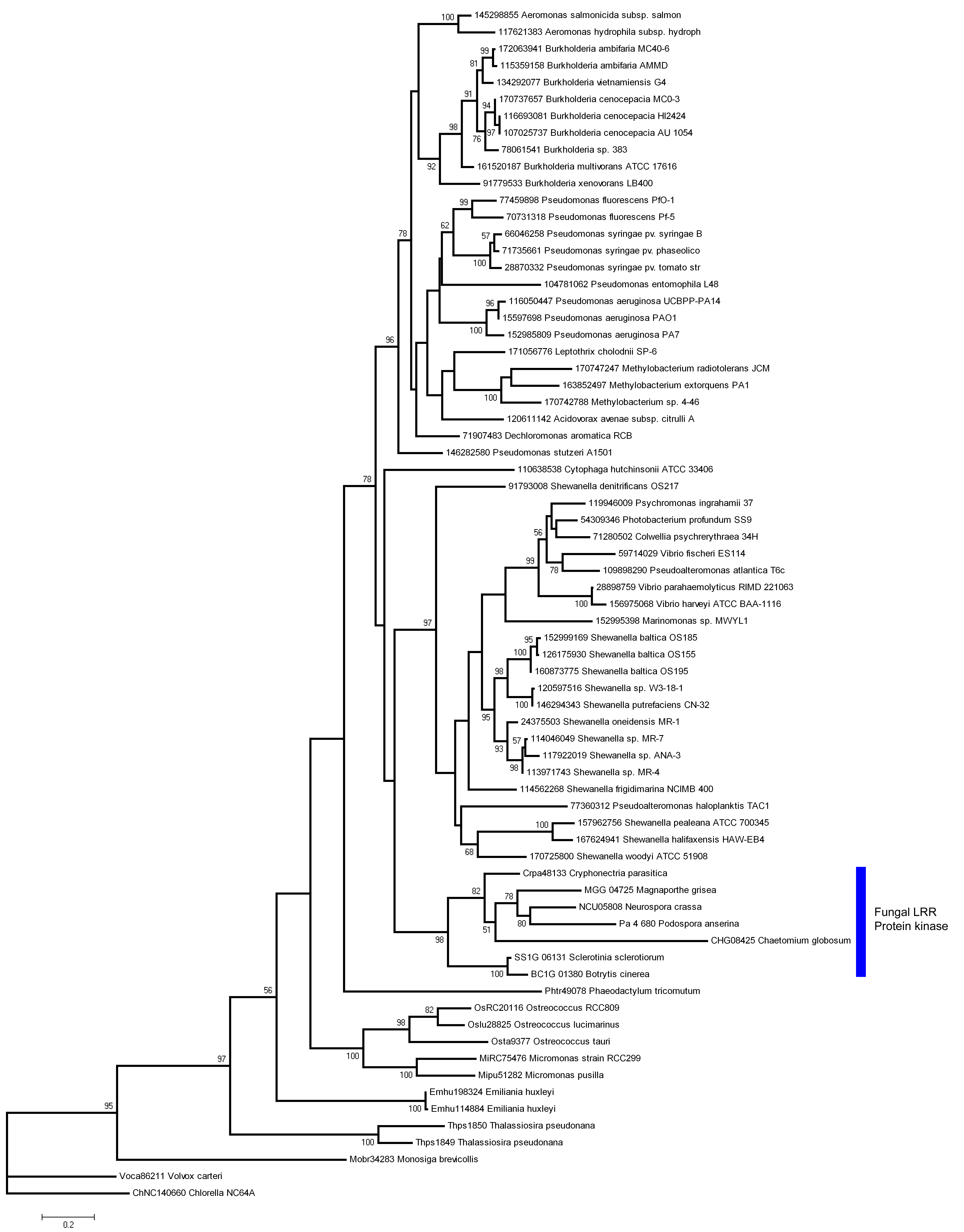

Supplement: Figure S1 — Phylogenetic tree constructed using sequences that show the greatest homology to a set of LRR-protein kinases found specifically in a few species of filamentous ascomycetes. Bootstrap support scores of 50 or above shown for each branch. Taxa are labelled with protein IDs (for sequences analysed in this project) or GI numbers for other sequences from NCBI database. Fungal sequences are indicated by a blue bar. Note: CHG08425 from Chaetomium globosom has an LRR domain, but lacks a protein kinase domain. (2.64 MB TIF) [file pone.0012725.s001.tif]

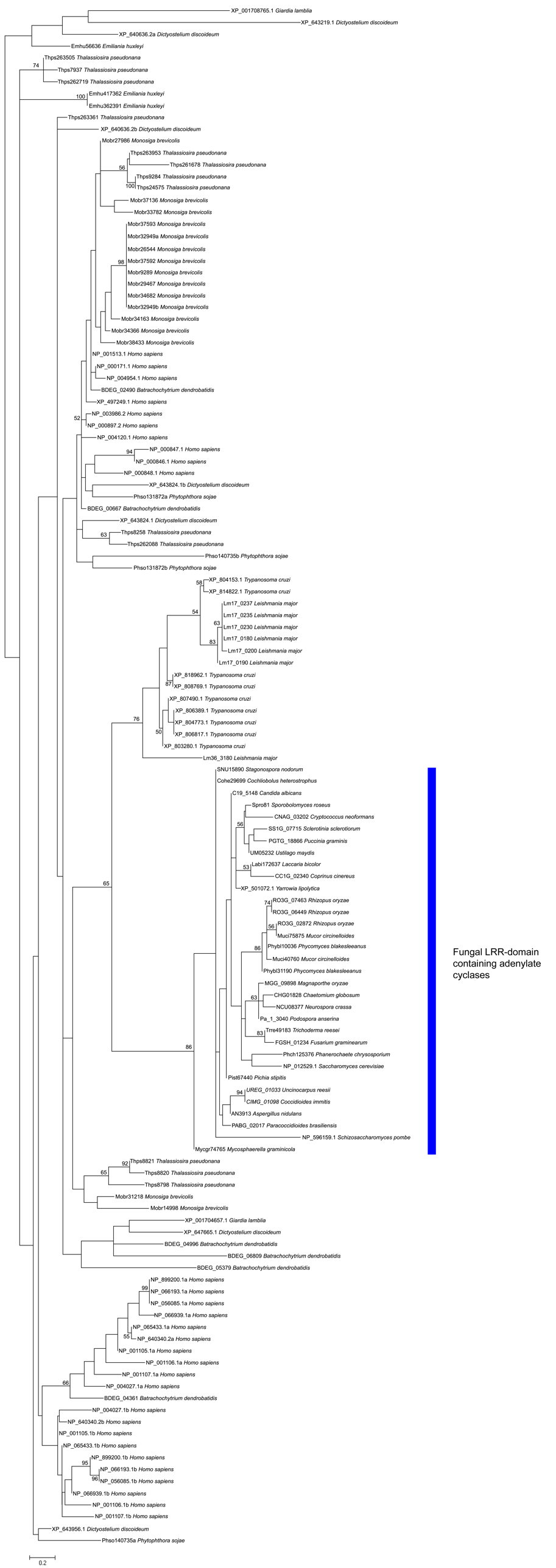

Supplement: Figure S2 — Phylogenetic tree constructed using adenylate/guanylate cyclase domains from representative species in each of the taxonomic groups used in this study. Bootstrap support scores of 50 or above shown for each branch. Protein IDs (from database sources listed in Table S1) are shown for each taxon. Fungal LRR-domain adenylate cyclases are indicated by a blue bar. (4.46 MB TIF) [file pone.0012725.s002.tif]

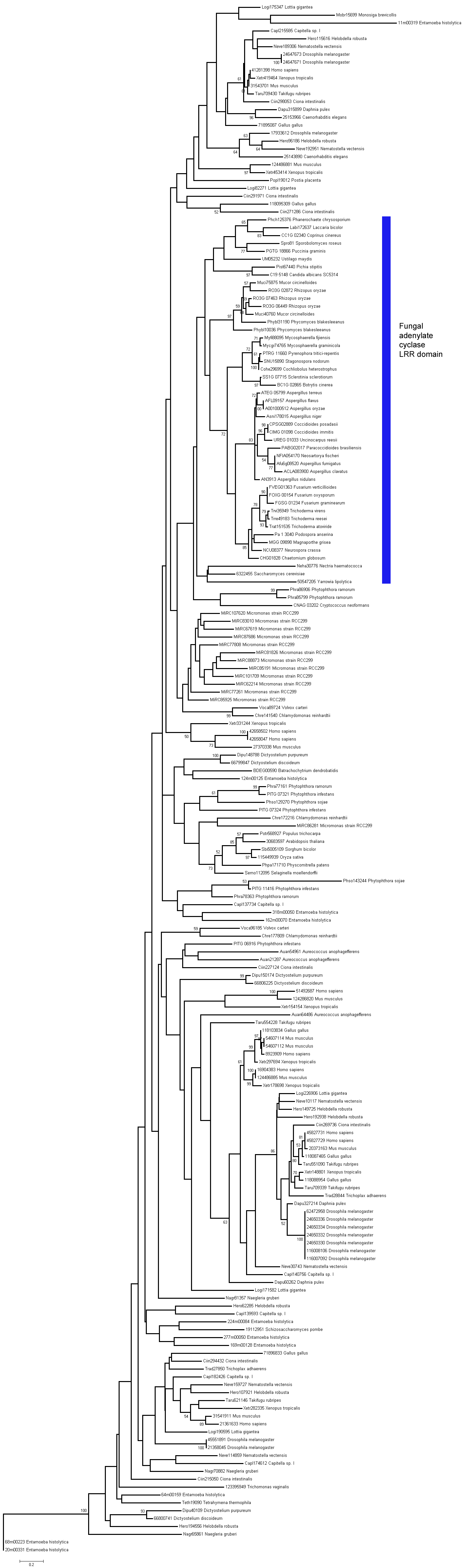

Supplement: Figure S3 — Phylogenetic tree constructed using LRR-domain sequences from ORTHOMCL1 cluster (see text for details). Bootstrap support scores of 50 or above shown for each branch. Protein IDs (from database sources listed in Table S1) are shown for each taxon. LRR-domains from fungal adenylate cyclases are indicated by a blue bar. (0.86 MB TIF) [file pone.0012725.s003.tif]
